# Breast Arterial Calcification on a Screening Mammogram: A Potential Cardiovascular Risk Stratification Tool in Women

**DOI:** 10.31083/RCM25958

**Published:** 2024-12-31

**Authors:** Ahmed Fathala, Deema Abunayyan, Leena Zeitouni

**Affiliations:** ^1^Department of Radiology, Nuclear Medicine and Cardiovascular Imaging, King Faisal Specialist Hospital & Research Center, 11211 Riyadh, Saudi Arabia; ^2^Department of Radiology, Medical Cities Program at the Ministry of Interior, Al Sahafa, 13321 Riyadh, Saudi Arabia; ^3^Department of Radiology, Women Imaging, King Faisal Specialist Hospital & Research Center, 11211 Riyadh, Saudi Arabia

**Keywords:** breast arterial calcification, coronary artery calcification, coronary artery disease, myocardial ischemia, screening mammography, atherosclerosis, breast cancer screening

## Abstract

Breast arterial calcification (BAC) is a common benign finding on a screening mammogram. Additionally, BAC is a type of medial calcification known as Mönckeberg medial calcific sclerosis, which differs from the intimal calcification seen in patients with coronary artery disease (CAD). Recently, BAC has appeared as a new cardiovascular risk stratification method. Studies have indicated a potential link between BAC and cardiovascular risk factors, particularly coronary artery calcification (CAC), as observed in coronary computed tomography. However, the association between BAC and myocardial ischemia and angiographic-proven CAD remains controversial. The usefulness of BAC during mammography as a potential screening tool for CAD has been the subject of uncertainty and debate for many years. This article reviews the current literature on BAC and its association with CAC, myocardial ischemia, and angiographic-proven CAD on both invasive and coronary computed tomography. Cardiovascular outcomes, current limitations, and future investigation and recommendations are also explored and discussed.

## 1. Introduction

Cardiovascular disease (CVD) is the leading cause of death in the USA and other countries [1]. Atherosclerosis is a common pathological process that leads to the occlusion of the coronary artery and other vessels. Atherosclerosis is a chronic inflammatory condition that eventually causes acute cardiovascular events due to plaque rupturing and thrombosis [2]. However, enough time exists for prevention because there is a lengthy latent period between the early stages of CVD and the clinical appearance of various clinical symptoms [3].

The Framingham Risk Score (FRS), a widely used tool for CVD risk stratification, is known to underestimate cardiovascular (CV) events [4]. Current data illustrate that up to 20% of all CV events occur without significant major risk factors [5]. Further, current guidelines recommend using risk factors-based algorithms to estimate the 10-year risk of atherosclerosis CVD (ASCVD) prevention [6]. However, these approaches also underestimate the presence and burden of coronary artery disease (CAD), leading to the underestimation of a large population of at-risk women [7]. Calculating the coronary artery calcium score (CACS) using noncontract electrocardiogram (ECG)-gated cardiac computed tomography has improved risk stratification in women compared with FRS [8]. Recently, breast arterial calcification (BAC) identified in screening mammography has garnered significant interest as a potential indicator of CVD and a noninvasive screening method for CAD. Numerous studies have investigated the association between BAC and coronary artery calcification (CAC) and cardiovascular outcomes in women [9, 10, 11, 12]. This article reviews current literature on the epidemiology, pathophysiology, and detection of BAC in screening mammography. Furthermore, we discuss the association between BAC and CAC, myocardial ischemia, and angiographic-proven CAD. In addition, we explore the occurrence of BAC in certain high-risk groups, such as patients with diabetes and chronic kidney disease (CKD). Finally, we discuss the current limitations and future investigations for BAC.

## 2. Methods 

All published research on the connections between BAC and CAC, CAD, myocardial ischemia, stroke, CKD, smoking, and diabetes was compiled to conduct this literature/narrative review. BAC, breast arterial calcification, mammary arterial calcification, Mönckeberg medial calcific sclerosis, intramammary arterial calcification, coronary artery disease, coronary artery calcium score, coronary computed tomography angiography (CCTA), invasive coronary angiogram, myocardial ischemia, single-photon emission computed tomography, CKD, and cardiovascular disease were the keywords used in the title, abstract, and subjects heading searches in the literature on the Ovid Medline and PubMed databases. The authors reviewed all English articles in their entirety. Data on the design and endpoint of each study, the prevalence of BAC, and the correlation between BAC and cardiovascular diseases were gathered, examined, and documented.

### 2.1 Epidemiology of BAC 

The most recent estimated prevalence of BAC in women undergoing screening mammography was 12.7% [13]. The most important determinate factor of BAC is age; BAC affects 10% of women in their 40s, whereas nearly half of women in their 80s experience it. Other important BAC-related factors include diabetes, parity, CKD, and CAD risk equivalents. BAC is common among patients with CKD, with an overall prevalence of 34.7%, and is associated with poor CV outcomes [14]. Race/ethnicity is another important factor affecting BAC. Hispanic women have the highest prevalence of BAC (34%), whereas Asian women have the lowest (7%). BAC occurs in 25% of African–American women and 24% of Caucasian women [15]. These findings are somewhat inconsistent with the prevalence of CAC in women in the MESA (Multi-Ethnic Study of Atherosclerosis) trial, where BAC occurred in 45% of Caucasians, 43% of Chinese women, and 37% of African–American women. However, these variations are probably caused by differences in population size among the studies [16].

### 2.2 Detection of BAC in Mammography 

BAC is distinguished in regular screening mammography by its characteristic appearance, known as genuine parallel linear calcification or tram-track, that parallels the vessel wall similarly to a railroad track.

**Fig. 1.**
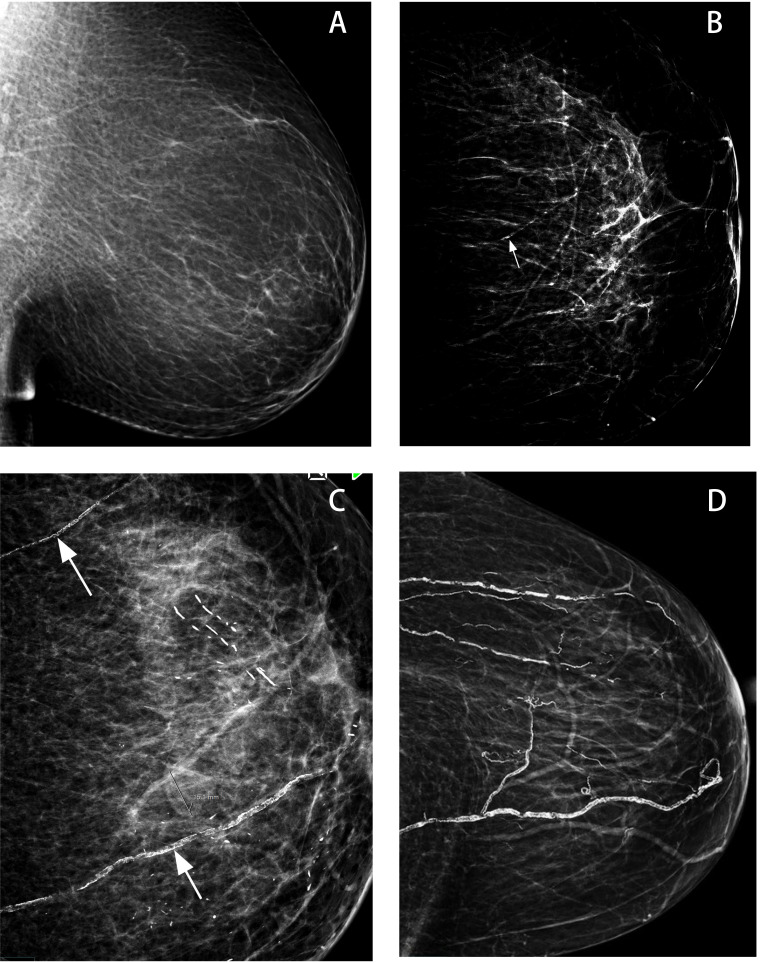
**Visual scoring of breast arterial calcification (BAC)**. (A) No BAC (scored 0). (B) Mild BAC: one vessel (arrow), single wall, and less than one-third of the vessel length (arrow). (C) Moderate BAC: two vessels (arrows), two walls, visualization of the lumen in the vessels, and less than two-thirds of the vessel length. (D) Severe BAC: multiple vessels, dense calcification with obliteration of the vessel lumen. Cited from reference [35].

BAC features a serpentine course rather than the typical branching pattern observed with other benign and malignant calcifications. However, calcification on one side of the vessel or within a small vessel wall can appear intraductal and present a diagnostic dilemma in mammography. Magnified views are often valuable in differential diagnoses for other causes of benign and malignant calcifications [17]. Based on the existence and degree of calcification, a 4-point scale can be used to rate the degree of BAC in mammography: (1) no vascular calcification; (2) little, punctate calcification without a tram-track sign or ring calcification; (3) coarse tram-track or ring calcification affecting less than three vessels; (4) severe calcification affecting three or more vessels (Fig. 1) [18].

A differential diagnosis of other benign liner breast calcifications includes secretory calcification entailing a smooth, thick, rod-shaped calcification that may feature diffuse linear branching, indicating its intraductal origin [19]. Sutural calcification occurs at or near the biopsy bed of an irritated breast and presents a characteristic tubular appearance [20, 21]. Other less common linear breast calcifications include filarial infection of the breast due to infection by *Wuchereria bancrofti* in endemic areas (e.g., South and Central America, the Caribbean, Africa, China, Southeast Asia, and Northern Australia). BAC related to filarial infections has a characteristic serpiginous, worm-like appearance [22]. In malignant linear calcification due to ductal carcinoma *in situ* and invasive ductal carcinoma, in addition to a fine-linear or linear branching appearance, the calcification of ductal carcinoma can be classified as grouped, pleomorphic, and dot–dash [23].

### 2.3 Pathophysiology 

In contrast to the intimal calcification seen in atherosclerotic illnesses, BAC involves vascular calcification within the breast and the medial layer of the vessel (Mönckeberg medial calcific sclerosis). Unlike advanced atherosclerosis, characterized by the accumulation of calcium phosphate ions in the vascular tissue, Mönckeberg calcification involves hydroxyapatite crystals deposited in the plaques [24]. Mönckeberg calcification is a frequent finding in screening mammography that does not always indicate a cancer risk [17]. The exact pathogenesis of Mönckeberg medial calcific sclerosis is unknown; no specific factors inciting the injury of vessel media have been identified.

### 2.4 Association between BAC and CAC 

Multiple observational studies have demonstrated an association between BAC and CAC; however, most of these are small, retrospective, and single-center studies (n = 100 to 500), except the cross-sectional study by Yoon *et al*. [25] that enrolled 2100 women with no symptoms who had digital mammography, coronary computed tomography angiography, and dual-energy X-ray absorptiometry performed. CAC and coronary atherosclerotic plaques (CAPs) were observed in 11.2% and 15.5% of the participants, respectively. Women with CAC and CAP showed a high prevalence and severity of BAC and low bone mass (LBM). The researchers concluded that BAC and LBM were substantially linked to the probability of subclinical CAD in women who did not exhibit any symptoms [25].

In a similar study with fewer subjects, Margolies *et al*. [26] studied 292 women using mammography and chest computed tomography within 1 year and evaluated CAC using semi-quantitative methods. BAC was associated with CAC and was superior to conventional cardiovascular risk factors in predicting CAC. However, the study failed to demonstrate a significant incremental value for BAC using the conventional risk stratification algorithm. In another study, Chadashvili *et al*. [27] found an association between CAC and BAC, with both chest computed tomography and screening mammography performed within 1 year and an association between BAC and a CACS >11. Furthermore, the authors demonstrated the positive predictive power of BAC for the development of CAD. In the positive-BAC group, 70% of women had a percentile >25, whereas only 45% of women in the BAC-negative group had a percentile >25. In contrast, Matsumura *et al*. [28] compared 98 women with BAC with a control cohort of 104 women without BAC and found that BAC was not predictive of a CACS >0. However, in an age-adjusted model, BAC demonstrated a significant correlation with high-risk calcium score, defined as Agatston Score >400.

In a meta-analysis of 927 women recruited in five studies, Abi Rafeh *et al*. [29] found that women with BAC detected using mammography had a 1.59-fold higher risk of angiographically diagnosed CAD. The authors concluded that there may be a greater chance of discovering obstructive CAD in coronary angiography if there is BAC on a mammogram [29]. Similarly, Hendriks *et al*. [13] studied the association between BAC and CV risk factors. BAC was associated with an increased risk of CV events and some known CV risk factors. The authors proposed that BAC might contribute to CV risk via different pathways from the intimal atherosclerotic process.

The association between BAC and hormonal therapy was assessed by Schnatz *et al*. [30], who evaluated the relationship between hormone therapy and breast cancer. Schnatz *et al*. [30] discovered that the prevalence of BAC was higher in menopausal women than in premenopausal women, suggesting that estrogen may impact the development of BAC. Previous hormone medication was substantially linked to a lower prevalence of BAC, even after controlling for age. Since there was a strong correlation between CV risk and hormonal balance in women both during and after the menopausal transition, the authors concluded that BAC may be a biomarker of sex-specific CV risk.

To investigate the role of BAC and CV risk beyond the aging process, Moshyedi *et al*. [31] assessed the association among BAC, CAD, and diabetes after adjusting for age. Subsequently, Moshyedi *et al*. [31] found that the presence of BAC indicated an additional risk factor for CAD in women aged <59 years (positive predictive value of BAC for CAD was 0.88; negative predictive value was 0.65). Similarly, in another cohort of women aged <60 years, there was a strong association between the presence of BAC on a mammogram and the presence of CAC on cardiac computed tomography [32].

Fathala *et al*. [33] evaluated the prevalence of BAC in screening mammography in Saudi women and its relationship to CV risk. Out of the 307 women who were enrolled, 46% had BAC; the women in the BAC-positive group were significantly older than those in the BAC-negative group. Additionally, significant correlations were found between BAC and diabetes, hypertension, and CAC but not between BAC and dyslipidemia, tobacco use, or a family history of CAD [33]. Except for the study by Moradi *et al*. [34], several other studies found a significant relationship between BAC and CAC; however, the variation in the study results and conclusions could be related to multiple variables such as small population size, possible selection bias, and different methods of evaluation BAC and CAC.

### 2.5 Association between BAC and Myocardial Ischemia 

Few studies have investigated the relationship between BAC in mammography and myocardial ischemia. Fathala *et al*. [35] conducted a cross-sectional retrospective study including 435 women who underwent screening mammography and stress myocardial perfusion imaging (stress MPI) within 1 year of each other. The mean age was 58 ± 8 years. Women with positive BAC were significantly older than women with negative BAC. A strong association was observed between BAC and hypertension, diabetes, and CKD, but none between BAC and dyslipidemia, smoking, and family history of CAD. Furthermore, no association was observed between BAC and myocardial ischemia on stress MPI. Myocardial ischemia was observed in 13% of women with BAC, with no significant difference with BAC-negative women [35]. Shobeiri *et al*. [36] performed a cross-sectional study on 400 women who underwent screening mammography and stress echocardiography to evaluate for myocardial ischemia. BAC was observed in only 15.2% of women, and the mean age in the positive group was significantly higher than in the negative group. A positive association was found between BAC and myocardial ischemia in the BAC-positive and BAC-negative groups (24.6% *vs*. 8.5 %, respectively; *p* < 0.001). Women with myocardial ischemia were more likely to have diabetes, hypertension, hyperlipidemia, and a history of CVD [36]. The discrepancy between these two studies is probably due to different population risk factors and inclusion and exclusion criteria. Currently, determining the relationship between BAC and myocardial ischemia using different noninvasive modalities for assessing myocardial ischemia is difficult, and further studies are needed.

### 2.6 Association between BAC and Coronary Computed Tomography Angiography 

Few studies, mostly with contradictory findings, have examined the relationship between BAC using mammography and obstructive coronary artery disease using CCTA, coronary artery stenosis severity classification was reported per Coronary Artery Disease Reporting and Data System (CAD-RADS) [37]. Kelly *et al*. [38] examined the association between BAC and CCTA findings within a cohort of women who underwent a breast-screening program. Of the 209 women who underwent CCTA, 104 also underwent mammography. BAC was a significant predictor for moderate coronary artery stenosis on the CAD-reporting and data system (CAD-RADS ≥3 disease) [38]. Even after binominal logistic regression analysis, BAC remained associated with CAD-RADS ≥3 disease. The authors concluded that BAC detected using mammography can predict obstructive CAD in symptomatic women [38]. In a comparable but smaller study, 100 women between the ages of 34 and 86 had both a mammogram and a CCTA. For both the mammogram and CCTA, a 4-point rating was applied. Using CCTA, 10 out of the 12 patients who had a moderate to advanced BAC on the mammogram also had moderate to severe CAD. The positive predictive value of BAC for CAD was 0.83 for the whole population, while the negative predictive value was 0.78. Following the CCTA, BAC presence was associated with CAD [18].

Unlike these earlier studies, McLenachan *et al*. [39] analyzed 405 women who had participated in the SCOT-HEART randomized controlled study, which assessed patients with suspected stable angina using CCTA and mammography. Visual evaluations were used to determine whether or not BAC was present in mammograms. Of the patients with BAC, 58 (62%; relative risk (RR) 1.26, 95% confidence interval (CI): 1.04, 1.53; *p* = 0.02) had CAC, 58 (62%; RR 1.27, 95% CI: 1.04, 1.54; *p* = 0.020); 19 (20%) had obstructive CAD (RR 1.62, 95% CI: 0.98, 2.66; *p* = 0.058). Although patients lacking BAC had a 95% chance of not having CAC, BAC had a low diagnosis accuracy for CAD [39]. The difference in the results between these studies is likely due to differences in demographics between the study populations.

### 2.7 Association between BAC and CAD on Invasive Coronary Angiography

Reports on the association between BAC and CAD on invasive coronary angiography (ICA) are mixed. Zgheib *et al*. [40] investigated the association between BAC on a mammogram and CAD in 104 women who underwent ICA and screening mammography; the mean age of the women with BAC was 72 ± 9.8 years, which was significantly higher than the mean age of the patients without BAC. However, no correlation was found between BAC and coronary angiography-proven CAD, even when severity was considered [40]. Furthermore, no correlation was discovered between BAC and angiographic-proven CAD in a recent retrospective analysis by Fathala *et al*. [41] involving 203 Saudi women who had ICA and mammography procedures performed within six months of one another. However, BAC, age, and several other traditional CAD risk variables strongly correlated. In a comparable retrospective study by Henkin *et al*. [42], 319 women between 50 and 70 years of age were enrolled, of whom 87 developed CAD, while 132 had normal ICA. In the CAD group, the prevalence of BAC was slightly greater (43.9 *vs*. 37.1, respectively; *p* = 0.138). The presence of BAC did not distinguish the individuals with angiographic indications of CAD from those with normal ICA. In contrast, Fiuza Ferreira retrospectively evaluated BAC using mammography and the ICA of 131 women aged 42–81 years [43]. In total, 85 women had CAD (41 with BAC and 44 without), while 46 had normal ICA (11 with BAC and 35 without). Furthermore, a strong association was found between BAC and CAD. One plausible reason for the disparity observed amongst earlier research is that the genesis of CAD in females involves factors beyond the mere formation of an obstructive lesion within the coronary circulation.

### 2.8 BAC and CV Outcomes

Due to increased arterial stiffness, BAC appears linked to an increased risk of CV events and may indicate the onset of medial calcification in other vascular arteries [44, 45].

Since blood flow properties are altered, atherosclerosis is worsened by the stiffening of the major arteries. Moreover, the reduced distensibility of the veins raises blood pressure, causing vascular remodeling and damage, eventually resulting in ischemia through concomitant atherosclerosis. In a population-based breast cancer screening initiative in the Netherlands from 1975 to 1977, 12,239 women aged 50 to 68 years showed a correlation between BAC and cardiovascular mortality, according to a longitudinal study by Kemmeren *et al*. [46] that evaluated the link between BAC and CV outcome. When age, diabetes, hypertension, and other factors were considered, the hazard ratios for mortality from CVD, mortality from CAD, and overall mortality were 1.29 (95% CI: 1.01–1.66), 1.44 (95% CI: 1.02–2.05), and 1.29 (95% CI: 1.06–1.58), respectively, in women with BAC detected using screening mammography compared with women without BAC [46]. Similarly, Iribarren *et al*. [9] reported hazard ratios of 1.32 (95% CI: 1.08–1.60) for CAD, and 1.44 (95% CI: 1.02–2.05) for ischemic stroke and 1.52 (95% CI: 1.18-1–98) for heart failure after adjusting for CV risk factors [9]. Hendriks *et al*. [45] recently reported a series of case–cohort studies within a longer prospective cohort study. The presence of BAC was significantly associated with CAD and combined CVD, including CAD, stroke, and peripheral arterial disease (PAD), for 12.2 years of follow-up after adjusting for conventional CV risk factors. In contrast to the previous studies, Abou-Hassan *et al*. [44] investigated the clinical implications of BAC in women with CKD. Here, BAC prevalence was 58% and was significantly associated with age, diabetes, and CKD duration. Both CAD and PAD were more common in patients with BAC than in those without.

### 2.9 BAC and CKD

Few studies have analyzed the occurrence and development of BAC in CKD at different phases of the illness. Additionally, knowledge of the relationship between BAC, abnormalities in mineral metabolism, and inflammation in CKD patients remains limited. To determine the prevalence, progression rate, risk factors, and clinical consequences of BAC in patients with CKD across the various disease stages in 310 women, Van Berkel *et al*. [14] performed a retrospective observational cohort study. The frequency of BAC was 34.7% and increased as CKD worsened. The characteristics of patients with BAC included higher age, a higher prevalence of CVD, a higher pulse pressure, and a slightly higher prevalence of diabetes. Compared to patients with CKD 5 recipients of renal transplants experienced a slower progression of BAC, which was linked to generally poorer CV outcomes [14]. Another retrospective study in patients with end-stage renal disease (ESRD) that enrolled 202 women found that BAC was significantly associated with age, diabetes, and ESRD duration. Both CAD and PAD were more likely to occur in patients with BAC [40]. Manzoor *et al*. [47] investigated the progression of BAC relative to CKD severity and found that BAC in CKD did not progress until the advanced stages of CKD, accelerated markedly in ESRD, and returned to the control rate after kidney transplantation. Furthermore, diabetes significantly increased BAC in patients with CKD and ESRD.

### 2.10 BAC and Peripheral Arterial Diseases 

Dale *et al*. [48] conducted a prospective study enrolling 645 women who underwent consecutive screening mammography and CT to detect benign vascular calcification. Dale and co-authors found a highly significant association between BAC on the mammogram and the presence of peripheral vascular calcification; they also reported that a lack of BAC correlated with a negative history of peripheral vascular diseases. Similarly, a prior study investigating the association between BAC and systemic vascular disease showed a statistically significant association between BAC and atheromatosis of the carotid or femoral arteries [49]. In addition, the relationship between BAC in mammography and carotid intima thickness (C-IMT) was studied in 100 women; the study demonstrated that BAC in mammography is independently associated with C-IMT apart from age and menopausal status [50]. Furthermore, a positive relationship was reported between BAC and brachial intima-media thickness [51]. The role of BAC as a marker for peripheral arterial disease is currently uncertain. Therefore, a larger prospective study of women without a history of peripheral arterial disease at bassline is required to determine whether incidental BAC in mammography detects peripheral vascular disease.

### 2.11 BAC and Diabetes 

The prevalence of BAC is high in patients with diabetes. Furthermore, a strong association between BAC and diabetes has been reported in many studies. Cetin *et al*. [52] found a 25.4% prevalence of BAC among women with diabetes and only 7% among patients with hypertension. The prevalence of BAC was observed to increase almost fourfold in patients with diabetes and threefold in patients with hypertension compared to controls with no diabetes or hypertension. The prevalence of BAC among patients with diabetes was even higher in other studies [52]. The prevalence of BAC was 40% in women with diabetes aged 45–68 years. The presence of BAC in menopausal women with diabetes aged >45 years was associated with microvacuolar chronic complications [53]. In addition, BAC has been associated with an increased risk of other diabetic complications (e.g., amputation, proteinuria, and retinopathy) [54, 55]. In a prospective study of women with and without diabetes, BAC was an independent risk factor for CV mortality (90% increase in cardiovascular mortality) [56]. Furthermore, a study found that the risk of diabetes was 4.5 times higher in women with BAC on a mammogram than in those without BAC [57]. However, other studies could not establish an association between BAC and diabetes [58, 59]. In conclusion, the question of whether the presence of BAC in mammography could be used to identify women at high risk for diabetes and CVD remains unanswered. Nevertheless, BAC has been suggested as a marker for CVD, as well as for diabetes.

### 2.12 BAC and Smoking 

Interestingly, several studies have demonstrated a null or inverse relationship between smoking and BAC [56, 60, 61]. However, the exact explanation of this inverse relationship between these two disorders has yet to be discovered. Smoking is a well-known risk factor for CVD [62]. One possible explanation of this inverse association may be that smoking-induced inflammation plays a role in BAC pathophysiology [63]. The MINERVA trial (multi-ethnic study of breast arterial calcium gradation and cardiovascular disease), one of the most recent and largest prospective studies, found no association between causal ASCVD risk factors (smoking and total cholesterol) and BAC [64]. Smoking causes CV events by increasing inflammation, thrombosis, and endothelial dysfunction but not vascular calcification [65, 66].

## 3. Limitations and Future Research 

This review has identified several studies that investigated the relationship between the presence of BAC in mammography and CAD. The overall relationship between BAC and CVD combines positive and negative associations. This mixed result is most likely because most studies were small, retrospective, and single-center, with possible selection bias and different demographics included among the studies. Although some studies indicated an association between BAC and CVD, uncertainty regarding the causality and pathophysiological mechanism remains. The pathophysiology of BAC may be mediated by osteogenic regulatory genes, similar to that observed in bone formation [12, 67], which contrasts with intimal artery calcification observed in CAC induced by macrophage activation, lipid deposition, and inflammation. The pathophysiologic processes involved in BAC and CAC indicate varying usefulness as markers for CVD. In addition, several studies demonstrated that only 30% of BAC cases indicate obstructive CAD on CCTA and invasive coronary angiography [37, 38, 39]. Furthermore, most CV events occur among women without BAC, as shown in the MINERVA study, indicating that BAC is not a sensitive marker of CVD. Moreover, a lack of association between BAC and ASCVD risk factors suggests that the pathophysiology of BAC may be distinct from that of CAC, as demonstrated by the null or inverse relationship between BAC and smoking and the inconsistent relationship between BAC and cholesterol level reported in various studies [13, 26]. Furthermore, the effects of statins on BAC have yet to be established.

Nevertheless, BAC is still useful for early-stage CVD risk stratification, although current evidence is based mostly on retrospective studies with inconsistent findings. Therefore, future prospective studies with established cardiovascular outcomes are crucial to confirm whether BAC improves CVD risk stratification beyond standard ASCVD risk models. A previous study to determine the attitudes of patients regarding mammographic reporting of BAC results, communications, and action revealed an overwhelming preference of patients to be informed about the presence of BAC on a mammogram [68]. Since data remain scarce on the utility of BAC for CVD risk stratification, guidelines need to be established on the manner of consultation with patients regarding the presence or absence of BAC in mammography [69]. Physicians must inform patients that zero CAC is not equivalent to low CVD risk and should not be used as a false assurance. However, while BAC in mammography does not necessarily indicate a high cardiovascular risk, it could be an opportunity to discuss other conventional CVD risk factors and optimize cardiovascular health [70].

## 4. Conclusions

Although BAC is a benign finding in standard screening mammography, there is a substantial correlation between BAC and CVD, according to multiple studies. The majority of published data indicate that BAC is associated with CAC. However, the association between BAC and myocardial ischemia and angiographic-proven CAD has not been unequivocally established. BAC is not associated with some traditional risk factors for coronary atherosclerosis, such as smoking and high cholesterol, suggesting a different pathophysiology. BAC is common in certain populations, such as diabetes and CKD, and is associated with more complications and poorer outcomes. Hence, BAC-validated qualification and routine reporting on routine mammography are encouraged. Moreover, future large-scale prospective studies of long-term outcomes are required to evaluate the potential clinical impact and cost-effectiveness of BAC in mammography. The presence or absence of BAC in mammography should initiate a physician–patient discussion regarding the modification and prevention of CVD risk factors. In the future, screening mammography could alter the course of the two most common causes of death in women, namely, breast cancer and CVD.
